# Assessing the extinction risk of insular, understudied marine species

**DOI:** 10.1111/cobi.13854

**Published:** 2021-11-12

**Authors:** Elin A. Thomas, Monika Böhm, Caroline Pollock, Chong Chen, Mary Seddon, Julia D. Sigwart

**Affiliations:** ^1^ Queen's University Marine Laboratory Queen's University Belfast Portaferry UK; ^2^ Institute of Zoology Zoological Society of London London UK; ^3^ Global Center for Species Survival Indianapolis Zoological Society Indianapolis Indiana USA; ^4^ Global Species Programme, Red List Unit International Union for Conservation of Nature (IUCN) Cambridge UK; ^5^ X‐STAR Japan Agency for Marine‐Earth Science and Technology (JAMSTEC) Yokosuka‐city Kanagawa Japan; ^6^ IUCN SSC Mollusc Specialist Group, Exbourne Okehampton UK; ^7^ Senckenberg Research Institute and Museum Frankfurt am Main Germany

**Keywords:** deep‐sea mining, hydrothermal vents, IUCN, marine, molluscs, threatened species, Especies amenazadas, UICN, marino, minería de mar profundo, moluscos, respiraderos hidrotermales

## Abstract

Hydrothermal vents are rare deep‐sea oases that house faunal assemblages with a similar density of life as coral reefs. Only approximately 600 of these hotspots are known worldwide, most only one‐third of a football field in size. With advancing development of the deep‐sea mining industry, there is an urgent need to protect these unique, insular ecosystems and their specialist endemic faunas. We applied the IUCN (International Union for the Conservation of Nature) Red List criteria to assess the extinction risk of vent‐endemic molluscs with varying exposure to potential deep‐sea mining. We assessed 31 species from three key areas under different regulatory frameworks in the Indian, West Pacific, and Southern Oceans. Three vent mollusc species were also examined as case studies of different threat contexts (protected or not from potential mining) to explore the interaction of local regulatory frameworks and IUCN Red List category assignment. We found that these assessments were robust even when there was some uncertainty in the total range of individual species, allowing assessment of species that have only recently been named and described. For vent‐endemic species, regulatory changes to area‐based management can have a greater impact on IUCN Red List assessment outcomes than incorporating additional data about species distributions. Our approach revealed the most useful IUCN Red List criteria for vent‐endemic species: criteria B and D2. This approach, combining regulatory framework and distribution, has the potential to rapidly gauge assessment outcomes for species in insular systems worldwide.

## Abstract 2

*Article impact statement* IUCN Red List assessments robustly compare extinction risk from deep‐sea mining at understudied hydrothermal vent habitats.

## Abstract (es)

Evaluación del Riesgo de Extinción de Especies Marinas Insulares Poco Estudiadas

**Resumen:**

Los respiraderos hidrotermales son oasis poco comunes en las profundidades del mar en donde se encuentran conjuntos de fauna con una densidad similar a la de los arrecifes de coral. A nivel mundial, sólo se conocen aproximadamente 600 de estos puntos calientes, la mayoría solamente del tamaño de un tercio de una cancha de fútbol. Conforme avanza el desarrollo de la industria minera en mares profundos, también hay una urgente necesidad por proteger estos ecosistemas únicos e insulares y a su fauna endémica especialista. Aplicamos el criterio de la Lista Roja de la UICN (Unión Internacional para la Conservación de la Naturaleza) para evaluar el riesgo de extinción que tienen los moluscos endémicos de los respiraderos ante una exposición variable a la potencial minería de mar profundo. Evaluamos 31 especies de tres áreas importantes bajo diferentes marcos regulatorios en los océanos Índico, Pacífico Occidental y del Sur. Analizamos tres especies de moluscos de los respiraderos como estudios de caso para diferentes contextos de amenazas (protegidas o no de la minería potencial) para explorar la interacción de los marcos regulatorios locales y la clasificación categórica dentro de la Lista Roja de la UICN. Descubrimos que estos análisis eran sólidos incluso cuando existía algo de incertidumbre en cuanto a la extensión total de la especie individual, lo que permite la valoración de especies que han sido descritas y nombradas recientemente. Para las especies endémicas de las chimeneas, los cambios regulatorios a un manejo basado en el área pueden tener un mayor impacto sobre los resultados de evaluación de la Lista Roja de la UICN que la incorporación de datos adicionales sobre la distribución de las especies. Nuestra estrategia reveló los criterios más útiles de la Lista Roja de la UICN para las especies endémicas de las chimeneas: los criterios B y D2. Esta estrategia, que combina un marco regulatorio con la distribución, tiene el potencial para medir rápidamente los resultados de las evaluaciones para las especies que se encuentran en sistemas insulares en todo el mundo.

## INTRODUCTION

The International Union for Conservation of Nature (IUCN) Red List of Threatened Species is the most comprehensive and rigorous tool for assessing the extinction risk of species worldwide (Rodrigues et al., 2006). It is widely used for conservation planning and to inform global policy (Hoffmann et al., 2008) because it allows the assessment of any taxon (except microorganisms) through standardized criteria (IUCN, 2012). More than 120,000 species have had their extinction risk evaluated under these criteria, but biases remain toward terrestrial species and vertebrates (IUCN, 2020).

Some researchers have questioned whether the quantitative thresholds of IUCN's Red List criteria could overestimate extinction risk for small‐bodied species, especially in combination with island or insular habitats, such as caves (Cardoso et al., 2011), karst mountains (Grismer et al., 2020), or coral reefs (Hobbs et al., 2010). Nonetheless, the IUCN Red List criteria have increasingly been used to classify extinction risk for small invertebrates (Collen & Böhm, 2012; Collen et al., 2016) with levels of data deficiency and applicable threats that are comparable to those in assessments of vertebrate taxa (Collen et al., 2012). These invertebrate assessments tend toward higher prevalence of range‐based (criterion B) assessments (Collen et al., 2016), yet the use of criterion B still integrates additional information about threat processes, continuing declines of the species or habitat, or extreme fluctuations (IUCN, 2012).

Deep‐sea hydrothermal vents are insular, extreme environments, characterized by chemosynthesis‐based geothermal ecosystems that support local biomass comparable to coral reefs or tropical rainforests (Van Dover, 2000). Around 600 tiny hydrothermal vent sites are known worldwide, each < 0.1 km^2^, and the total global area is around 50 km^2^ (Sigwart et al., 2017; Van Dover et al., 2018). Vent communities are distinctly different from the surrounding deep‐sea benthos, exhibiting high endemism and regional variation in species composition, and many endemic species have only limited connectivity via the dispersal of pelagic larvae to other vents (Rogers et al., 2012; Yahagi et al., 2019).

The unique geological setting of hydrothermal vents is also characterized by high concentrations of metals, such as copper, nickel, cobalt, and zinc, and other targets of the emergent deep‐sea mining industry (Van Dover et al., 2018). The exploitation of seabed resources, including the majority of hydrothermal vent sites, is regulated by the International Seabed Authority (ISA) in areas beyond national jurisdiction or by individual countries when a site is in an exclusive economic zone (EEZ) (Thompson et al., 2018). Lifting tests for seafloor massive sulfides have already started in Japan (Okamoto et al., 2019). This is a rapidly developing industry for which commercial‐scale mining regulations are still being negotiated by the ISA, including the requirement for environmental impact assessments, and there is an urgent need for conservation tools to inform policy decisions (Dunn et al., 2018; Glover et al., 2018; Van Dover et al., 2018; Sigwart et al., 2019).

Among more than 700 species known from vents, molluscs account for approximately one‐third of known taxa (Chapman et al., 2019). Most are endemic species not found in any other ecosystems, including other chemosynthesis‐based habitats (Wolff, 2005). From the scaly‐foot snail (*Chrysomallon squamiferum*) with iron‐infused scales to giant vesicomyid clams with red blood, vent molluscs span five taxonomic classes and display an amazing array of diversity (Chapman et al., 2019). Red List assessments for other molluscs in terrestrial, freshwater, and marine habitats include some with restricted ranges (Clements et al., 2008; Neubert et al., 2019), as well as widespread long‐lived species in decline (Cuttelod et al., 2011; Lopes‐Lima et al., 2019). Molluscs endemic to vents are range restricted and predominantly data poor because knowledge is limited by sampling logistics in remote, extreme environments. Furthermore, vent‐endemic species are restricted, not only to hydrothermal‐vent environments, but also to vents in specific ocean basins or regions (Rogers et al., 2012).

Recent research has highlighted the lack of conservation capacity for neglected biodiversity, particularly in areas of high endemism (Hochkirch et al., 2020). Consistent application of IUCN Red List criteria is challenging for understudied, range‐restricted species and has previously led to variation in assigned extinction‐risk categories, even between species with near‐identical distributions and habitats (Munzinger et al., 2008). With urgent conservation now required for deep‐sea areas under threat from mining, we recognized the need to establish and ensure a reliable but rapid approach to IUCN Red List vent‐endemic species.

We applied IUCN Red List criteria to vent‐endemic species to combat data deficiency, accurately assess extinction risk, and ensure the consistency of assessment outcomes in insular vent ecosystems. These assessments of vent‐endemic molluscs included the entire deep‐sea mining threat range, from protection against mining to exploratory and exploitative mining licenses, and further provide a comparative basis to understand the interaction of species biology, range, and regulatory measures on assessment outcomes. We selected three assessments for species in different threat contexts as case studies to explore the interaction between regulatory frameworks and IUCN Red List assessment outcomes and demonstrate how the assigned extinction‐risk category is predicted to shift under hypothetical changes to deep‐sea mining regulations and conservation policies. Our novel approach for applying the IUCN Red List criteria to vent specialist species may have potential for wider application to other poorly known endemic species across a range of global ecosystem types.

## METHODS

Thirty‐one of 184 vent‐endemic molluscs described to date from global oceans were selected to test the applicability of the IUCN Red List criteria and compare different regulatory frameworks that govern conservation and mining activities. The key areas were identified as the Indian Ocean, where the ISA has signed several exploratory mining contracts with various sponsoring agencies, the West Pacific Ocean, where many vent fields lie within EEZs so that the granting of mining licenses is controlled independently by each national government, and the Southern Ocean, where a large proportion of vent fields are protected from exploitation by the Antarctic Treaty. We then compared these assessments to determine which variables had the largest influence on the assessment outcome. We further examined specific case studies to determine what impact a change in regulation might have on the assessment outcome.

Assessments of extinction risk for the IUCN Red List first require examining the applicability of the five standard criteria (IUCN Standards & Petitions Committee, 2019) given the data availability for hydrothermal vent molluscs (Table 1). For most vent‐endemic species, there are no reliable data on population sizes or trends (criteria A, C, and D/D1). Because commercial deep‐sea mining is still in development, there is also no realistic way to quantify extinction probabilities if mining were to commence (criterion E, IUCN, 2012). These assessments were thus entirely reliant on criteria B and D2, which identify species with restricted distributions that are currently, or at risk of, experiencing significant decline. Based on this information, species are then assigned to an IUCN Red List category, ranging from least concern (LC) with low risk of extinction, to increasing threat levels (near threatened, NT; vulnerable, VU; endangered, EN), up to critically endangered (CR) with extremely high risk (IUCN, 2012). When there are inadequate data available, a species may be assessed as data deficient (IUCN, 2012).

**TABLE 1:** Applicability of the five standard International Union for Conservation of Nature's Red List criteria to species endemic to deep‐sea hydrothermal‐vent environments (IUCN Standards & Petitions Committee, 2019)

Criterion	Description	Suitability for vent species	Applicable
A	Population size reduction	There are currently no population estimates for most vent‐endemic species. There is no basis for estimating temporal trends because many vent fields have been observed only a few times.	No
B	Geographic range	Hydrothermal vents are extremely spatially restricted, and the majority of vent species are endemic. Data are available for the geographic range of vent species, including number of locations. It is possible to estimate the area of occupancy and extent of occurrence for vent‐endemic species.	Yes
C	Small population size and decline	Vent‐endemic species are typically locally abundant but highly restricted to small areas within an already small habitat. Abundance has never been quantified in a way that would provide the data necessary to assess risk with this criterion.	No
D	Very small or restricted population	There are no data available for the number of mature individuals (D/D1). Under the same principles as for criterion B, there are data available for the restricted ranges of vent species (D2).	D2 only
E	Quantitative analysis of extinction probability	Deep‐sea mining licenses can span several hundreds of kilometers, and there is currently little to no information available regarding the time scales and extent of proposed mining operations.	No

Although the full distribution for some species may not yet be known, assessments must be based on the best available data at the time of assessment, and the currently known area of occupancy (AOO) and extent of occurrence (EOO) provide important additional context. To clarify specific IUCN Red List terminology relevant to criteria B and D2 in the assessment of extinction risk of vent species, we have provided brief explanations of our application approach.

### Location

The IUCN defines *location* as a distinct area where a threatening event can rapidly affect all individuals in the area; thus, the size of a location depends on the area covered by the threatening event (IUCN, 2012). For example, locations may be defined by the predicted or previously observed extent of the threat impact (e.g., lava flow or fire paths) or may be defined based on the size of an area over which similar regulations apply (e.g., in the case of harvesting or collection of a species [IUCN Standards & Petitions Committee, 2019]). A location is not a measure of the number of subpopulations. Because deep‐sea mining is the single greatest threat to vent‐endemic species, mining regulatory frameworks and scale of mining impacts should form the basis for defining a location in this context.

Although human activity may only affect a portion of a species’ population in other systems, deep‐sea mining is projected to have an adverse and potentially irreversible impact on vent species (Niner et al., 2018), affecting all individuals within proximity of a mining event. There is a limited literature on the extent of mining impacts; however, current evidence suggests that sediment plumes produced by mining activity have the potential to affect areas up to 70 km from the mining site (Luick, 2012). Vent habitats are small, isolated, and characterized by high levels of endemism, so the impacts of sediment plumes from mining cannot be realistically compared with similar impacts in nearshore environments. In line with the IUCN's precautionary principle (Cooney, 2004), an 80‐km minimum threshold to separate vent fields into different locations ensures that a mining event at one location will not affect another. Therefore, a location encompasses all vent fields within a prescribed management area with a biologically and geologically coherent identity and within 80 km of each other.

Although this is the established standard at present, there remains an element of uncertainty around the 80‐km threshold owing to the lack of information available on the spread of deep‐sea mining sediment plumes. We, therefore, anticipate that this distance could be adjusted in future assessments, as new modeling data on the impact of mining at hydrothermal vents become available. Such adjustments would allow the threshold for location to be amended for sediment characteristics, depth, and current strength within specific regions.

### Area of occupancy

Single hydrothermal vent fields are consistently < 0.1 km^2^, and vent‐endemic species are rarely found in more than 10 different vent fields (Sigwart et al., 2017). The IUCN recommends calculating *AOO* with a 2 × 2 km (4 km^2^) grid (IUCN, 2012; Keith et al., 2018; IUCN Standards & Petitions Committee, 2019). This has been a contentious issue across different taxonomic groups (Cardoso et al., 2011; González‐Mancebo et al., 2012; Breiner & Bergamini, 2018); however, the importance of consistency supersedes the need for a more specific estimate of AOO for hydrothermal‐vent species. We, therefore, applied a 4 km^2^ buffer area around each vent field occupied by the species and the sum of these areas was the AOO. Though unlikely in a vent scenario, if multiple vent fields lie within the same 2 × 2 km cell, they should be recorded as a single 4 km^2^ AOO.

### Extent of occurrence

The *EOO* encompasses all known, inferred, or projected sites of present occurrence of a species and is estimated using a minimum convex polygon (IUCN, 2012; Joppa et al., 2016). The EOO is intended as a measure of the spatial spread of a species’ extinction risk; a large EOO reflects a lower probability that the entire population will be affected by a single threatening event (IUCN, 2012). The endemism of vent species means that they are likely unable to colonize other deep‐sea ecosystems within their range, often resulting in large expanses of seafloor between vent fields that are included in a species’ EOO. A species occurring in two or more widely separated vent fields (large EOO) may encounter different threat levels (e.g., one field may be protected, another under mining concession), resulting in a lower extinction risk, whereas a species occurring in two or more vent fields geographically close to each other (small EOO) may be affected by the same mining concession.

### Continuing decline or plausible threat

The IUCN definition of *continuing decline* is “a recent, current or projected future decline (which may be smooth, irregular or sporadic) which is liable to continue unless remedial measures are taken” (IUCN Standards & Petitions Committee, 2019). For criterion B, a continuing decline may be observed, estimated, inferred, or projected in EOO, AOO, extent or quality of habitat, or number of subpopulations, locations, or mature individuals (IUCN, 2012). For criterion D2, a *plausible threat*, if realized, will rapidly lead to a species becoming CR or extinct (EX). We determined continuing decline and plausible threats based on deep‐sea mining regulations. A plausible likelihood of mining commencing exists in the areas of exploratory contracts signed by the ISA or in the EEZ of a nation that has not implemented a deep‐sea mining moratorium. By contrast, effective protection from mining (e.g., mining moratorium and marine protected area [MPA]) means that there is neither a continuing decline nor a plausible threat present. Such regulations are only considered effective where the threat is sufficiently mitigated by their implementation, for example, MPAs that include specific legislation preventing seabed mining (Kelleher, 1999).

## RESULTS

**FIGURE 1:**
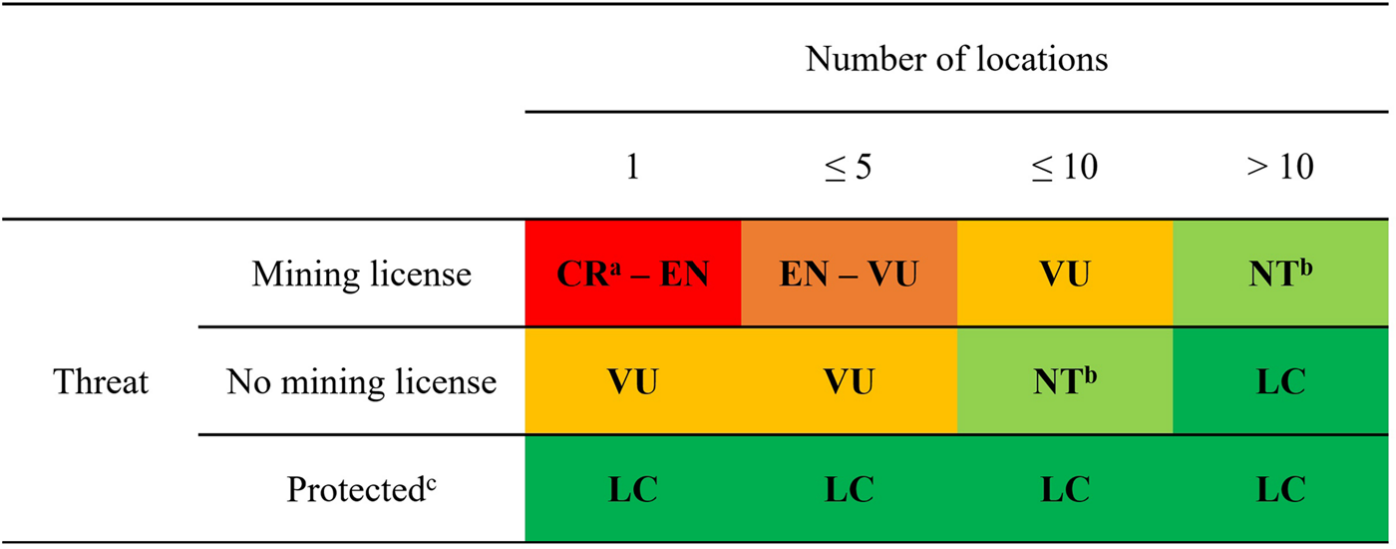
Relationship between locations of and threats to hydrothermal vent‐endemic molluscs and the outcome of their International Union for Conservation of Nature Red List assessments. Abbreviations: CR, critically endangered; EN, endangered; VU, vulnerable; NT, near threatened; LC, least concern. ^a^Assessed as CR (possibly extinct) where there is active commercial mining; ^b^Species were also assessed as NT where there are both designated mining licenses and protected areas located within their range or where the species are not protected across their entire range; ^c^Protected status refers to any established conservation measure that specifically protects a species from mineral extraction (e.g., local or regional marine protected area designation or mining moratorium)

Among the 31 assessments published by July 2020, 27 vent‐endemic species were listed as threatened by deep‐sea mining (Appendix S1). Of the 27 species listed under threatened categories, 17 were assessed under criterion B, 9 under criterion D2, and 1 under both criteria B and D2 (Appendix S1). The status of these species is closely tied to both the number of known locations for each species and the local regulatory frameworks, which we illustrated as a matrix combining location and threat (Figure 1). Five CR species were known only from single locations within exploratory mining contract areas in the Indian Ocean, signed by the ISA to either the Chinese Ocean Minerals Resources Research and Development Association (COMRA) or the Federal Institute for Geosciences and Natural Resources of Germany (BGR) (ISA, 2021). The four species assessed as LC were restricted to areas of the Southern Ocean protected by the South Georgia and the South Sandwich Islands (SGSSI) MPA and the Madrid Protocol of the Antarctic Treaty.

### *Gigantopelta chessoia* Chen, Linse, Roterman, Copley & Rogers, 2015 (case study 1)

**FIGURE 2:**
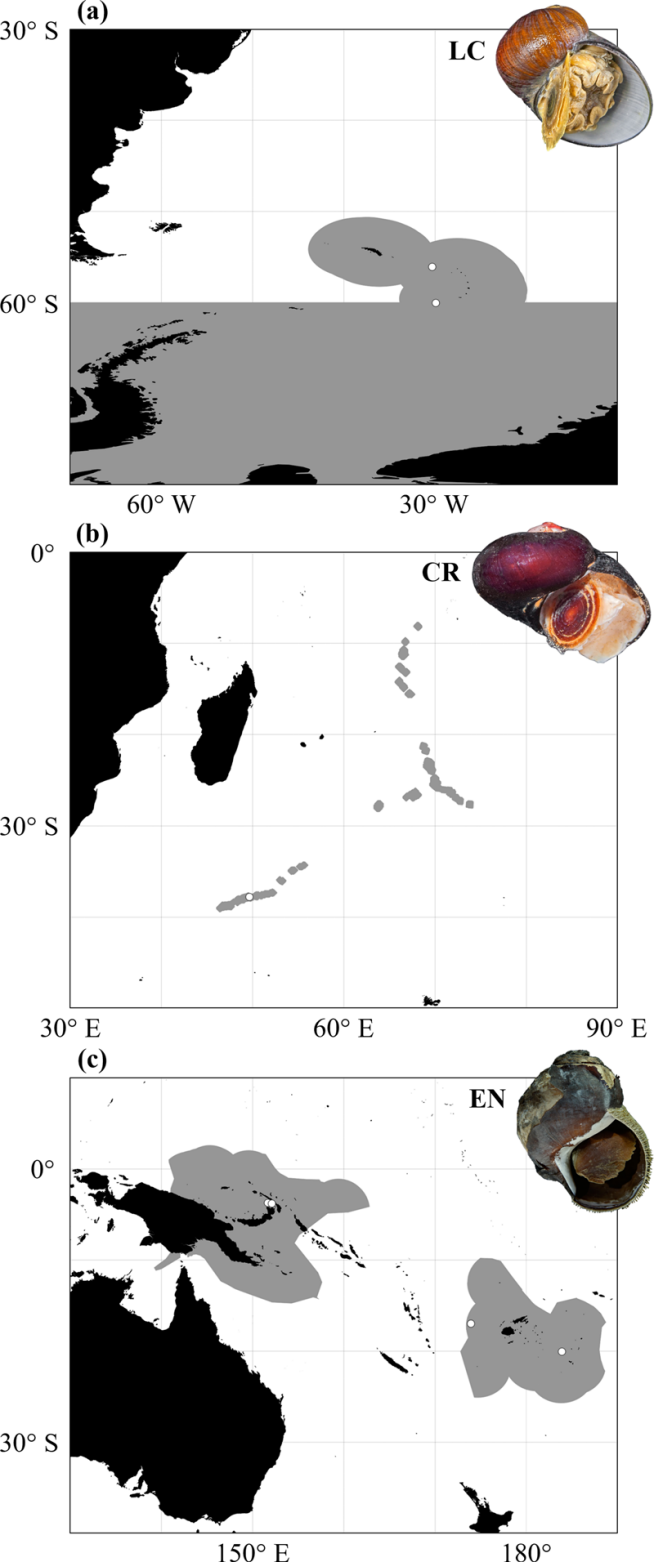
Geographic distribution (white dots) for the three case study species: (a) *Gigantopelta chessoia*, hydrothermal vent fields on the East Scotia Ridge (gray, area protected by the South Georgia and the South Sandwich Islands marine protected area and the Madrid Protocol of the Antarctic Treaty), (b) *Dracogyra subfusca*, Longqi hydrothermal vent field on the Southwest Indian Ridge (gray, mining contracts for polymetallic sulfide exploration signed by the International Seabed Authority), and (c) *Alviniconcha boucheti*, hydrothermal vent fields in the southwestern Pacific (gray, exclusive economic zones of Papua New Guinea, Fiji, and Tonga). Abbreviations: CR, critically endangered; EN, endangered; LC, least concern

Endemic to two hydrothermal‐vent fields on the East Scotia Ridge in the Southern Ocean, *Gigantopelta chessoia* is listed as LC owing to the significant protection from the threat of deep‐sea mining in these localities (Thomas et al., 2020) by the Antarctic Treaty and new legislation enacted within the SGSSI MPA prohibiting all mineral exploitation (Linse et al., 2019) (Figure 2a). The consequent lack of anthropogenic threat for species endemic to these localities is key to the assessment of *G. chessoia* as LC, despite the species’ naturally limited range. The worst‐case scenario for this species would be removal of the MPA, dissolution of the Antarctic Treaty, and granting of mining rights, in which case the species could be reassessed as EN. Given the global geopolitics of the Southern Ocean, this is extremely unlikely (Dodds, 2010), and in the published assessment, we indicated that changes to allow mining in the SGSSI EEZ could move the species to near threatened under D2 because there would be a plausible future threat that could rapidly lead to an EN assessment for the species.

### *Dracogyra subfusca* Chen, Zhou, Wang & Copley, 2017 (case study 2)

The gastropod *Dracogyra subfusca* is endemic to a single hydrothermal vent field on the Southwest Indian Ridge (Figure 2b). The Longqi vent field spans an area only 100 × 150 m (Tao et al., 2014) and lies within an area of seabed under an exploration‐phase deep‐sea mining contract between the ISA and COMRA. This contract allows for experimental extraction and testing, so activity conducted under the current contract may already have had an effect or may in the near future. As a result of this threat, an associated projected continuing decline, and the small area in which the species is found, *D. subfusca* is currently listed as CR (Thomas et al., 2019a). If the ISA were to allow exploitative mining operations to commence at the Longqi vent field, the impact would be catastrophic for its endemic fauna, including the removal of all known habitat currently occupied by *D. subfusca*. Such an event would prompt the reclassification of *D. subfusca* as CR (possibly extinct), a classification to identify species that are likely extinct but for which confirmation is required (Butchart et al., 2006). From its original description in 2017, there is a possibility that this monospecific genus may be extinct within few years of its discovery, owing to human activity.

### *Alviniconcha boucheti* Johnson, Warén, Tunnicliffe, Van Dover, Wheat, Schultz & Vrijenhoek, 2014 (case study 3)

Hydrothermal vent‐endemic species also have the potential to be downlisted if appropriate conservation is established and maintained. *Alviniconcha boucheti*, known from four vent fields within the EEZs of Papua New Guinea, Fiji, and Tonga (Figure 2c), is currently assessed as EN based on the threat imposed by Nautilus Minerals’ Solwara 1 deep‐sea mining project in the waters of Papua New Guinea (Thomas et al., 2019b). However, following the collapse of this mining venture in 2019, a 10‐year moratorium on deep‐sea mining was proposed by Fiji at the annual Pacific Islands Forum and was supported by Papua New Guinea (Kakee, 2020). Because the species’ assessment was published in 2019, a downlisting reassessment could not be published until 2024, providing that “none of the criteria for the higher category has been met for five years” (IUCN, 2012). If, by then, the Pacific Island countries were to impose a regional moratorium on deep‐sea mining until sufficient scientific and technological advancements were made to inform environmentally sound mining legislation, the vent‐endemic species known from this region, including *A. boucheti*, would be downlisted to LC.

## DISCUSSION

Hydrothermal vent ecosystems are populated by understudied species that are increasingly under threat. As demonstrated here for vent molluscs from three biogeographic regions, a list of endemic species from each vent site alongside their IUCN Red List status can serve as a proxy for their threat potential and biodiversity uniqueness, two important objective standards that are widely accepted and can be understood by a range of stakeholders (e.g., WWF, 2020). The location‐threat matrix (Figure 1) can be used to gauge assessment outcomes and could allow for more rapid assessment of additional vent‐endemic taxa, as well as other insular deep‐sea habitats threatened by resource extraction.

For deep‐sea species, as in other environments that are difficult to access, an overriding concern is whether there is a sufficient basis of evidence to judge the status of a population. This is a familiar problem in many other species that are cryptic, nocturnal, ground dwelling, arboreal, or simply occupy extremely remote areas (e.g., Trageser et al., 2017). Many methods that are used to supplement terrestrial observations, such as camera traps or indigenous knowledge (reviewed in Willcox et al., 2019), are not available in the deep sea, where observational opportunities are starkly limited. With the increasing development of advanced underwater imagery (e.g., Ocean Networks Canada), similar observational tools may become available to support future deep‐sea assessments. However, in this context, it is particularly interesting that we found that geographic distribution was sufficient to support an IUCN Red List assessment (criteria B and D2) and that regulatory changes to area‐based management (by influencing decisions on continuing decline or plausible threat) can have a greater impact on IUCN Red List assessment outcomes than additional data about species distributions.

The first hydrothermal‐vent endemic species to be added to the IUCN Red List, the scaly‐foot snail, was assessed as EN using criterion B (Sigwart et al., 2019). Since the date of that assessment, new records of the species have been reported by recent deep‐sea exploration cruises (Zhou et al., 2018; Jang et al., 2020; Sun et al., 2020a, 2020b), but a reassessment including these additional distributional records would still remain at EN. Although new records include a potential new location for the scaly‐foot snail, these vent fields also lie within ISA exploratory mining license areas and present the same established threat to the species. A change in regulation to protect a substantial portion of the population from the known threats, however, would merit reassessment within a different category.

If mining commences at a vent, the rapid impacts could mean that any endemic species will likely be extinct before they are listed in a more threatened category (Niner et al., 2018). A precautionary approach to IUCN Red List assessments provides greater opportunity for policy makers to establish appropriate protection for the species before disturbance, ensuring more proactive and preventative conservation rather than reactive “fire‐fighting” measures (Wilson et al., 2011; Walls, 2018; Le Breton et al., 2019). The implementation of effective conservation measures would lead to a genuine change in the IUCN Red List status of a species.

### Toward rapid assessment of remote systems

Our precautionary but realistic attitude, following IUCN Red List guidelines, clearly communicates the urgent threats posed by mining to fragile deep‐sea ecosystems. In species groups where population size and monitoring data are unavailable and assessment relies entirely on range data, it has often been argued that the IUCN Red List criteria may overestimate (e.g., island invertebrates [Cardoso et al., 2011]) or underestimate extinction risk (e.g., European butterflies [van Swaay et al., 2011]). For island species or species of insular habitats, overestimates are mostly due to restricted species range leading to small AOO or EOO values. Yet, AOO and EOO are not the sole requirements for criterion‐B‐assessed species; they must also meet two of three requirements for severe fragmentation or few locations, continuing decline, or extreme fluctuations (not relevant for vent molluscs) to be listed as threatened. For assessment as VU under criterion D2, the small AOO or few locations must be accompanied by a plausible future threat that could rapidly cause the species to become CR, or even extinct. Often, these subcriteria are neglected in studies on the impact of insularity on IUCN Red List categories (e.g., González‐Mancebo et al., 2012), whereas our location‐threat matrix integrates the number of locations with different scenarios of continuing declines, based on the granting of mining licenses or effective protection of hydrothermal vents. Furthermore, where uncertainty is prevalent, we stated the range of categories a species may qualify for and justify our final listing based on the relevant available information pertaining to threats. All our published assessments detail scenarios under which a species should be reevaluated in a different IUCN Red List category in the future.

The location‐threat matrix reflects all possible threat scenarios for deep‐sea mining. Although the matrix has only been informed by molluscs, it is applicable to all hydrothermal vent‐endemic species. Most vent areas are extremely small, usually in the order of 0.1 km^2^, and even with a 4‐km^2^ buffer for each site, the threatened category thresholds for AOO under criteria B or D2 are always met by vent‐endemic species. The small spatial scale and the dependence of vent species on the unique geochemical setting mean that mining these areas would produce catastrophic impacts for endemics. This contrasts with other types of habitats, where differing sensitivities to threats, such as pollution or turbidity, could influence the assessment outcome. In this case, the anticipated impact of mining would be destruction of all local habitat. Based on the restricted range of these habitats, the likelihood that species occur only at few sites, and the impacts of potential local mining, there is no scenario where a species is located within a mining license area and would be assessed as LC.

Our location‐threat matrix can be used to address the need for a rapid approach to identifying and prioritizing IUCN Red List assessments for species under the greatest threat (Le Breton et al., 2019; Liu et al., 2020). Furthermore, by committing to applying Red List criteria as intended by the IUCN (e.g., using 4 km^2^ buffer for AOO), our approach ensures consistency between vent Red List assessments and enables the comparison of extinction risk across vent species and regions. Consistency in IUCN Red List assessments is imperative to support objective decision‐making for species conservation. Ensuring this consistency is a core value of the IUCN Red List (Mace et al., 2008), and strict guidelines ensure reliability in its global application (Rodrigues et al., 2006). Although others have suggested modifications to refine IUCN Red List criteria for specific groups (e.g., Cardoso et al., 2011; González‐Mancebo et al., 2012), we demonstrated that it is possible to efficiently and accurately assess the extinction risk of even the most data‐limited, endemic species with geographic range criteria.

### Applying the location‐threat matrix

With all ∼600 global vent fields occupying a total area of only ∼50 km^2^ (Sigwart et al., 2017; Van Dover et al., 2018), it is easy to understand why the AOO and EOO parameters as defined by IUCN are sometimes criticized (Cardoso et al., 2011; González‐Mancebo et al., 2012; Breiner & Bergamini, 2018). For example, at the time of assessment, *Bathymodiolus manusensis* Hashimoto & Furuta, 2007 was known from a single location with projected continuing decline caused by the mining threat to that location. This location encompasses three different vent fields within 80 km of each other; thus, the estimated EOO (171.23 km^2^) and AOO (12 km^2^) exceed the respective 100 and 10 km^2^ thresholds for CR under criteria B1 and B2. Consequently, *B. manusensis* was assessed as EN rather than CR (Thomas & Sigwart, 2020).

This example demonstrates the robustness of our findings in the face of uncertainty in species identification and distribution data. After the assessment of *B. manusensis*, a new study reported another locality for this species (Lee et al., 2021), additional to previous records with tentative or uncertain identification that could not be considered within the published assessment (Miyazaki et al., 2010). Yet, including these additional localities would not alter the EN assessment based on our location‐threat matrix approach, owing to the enforcement of the AOO and EOO parameters. The location‐threat matrix allows accurate assessment of species extinction risk, even in the face of uncertainty.

Uncertainty in estimates of AOO, EOO, or factors encapsulated in the subcriteria, and the risk tolerance adopted toward this uncertainty (e.g., evidentiary vs. precautionary), can affect IUCN Red List assessments (Akçakaya et al., 2000; Romeiras et al., 2016). Yet, the extra buffer included in calculating the AOO in this way does in fact reflect the potential limits of a single mining event to affect the entire population of the species at the individual sites within this location. This emphasizes the importance of careful consideration when applying the IUCN Red List criteria, particularly when using methods for rapid assessment (Le Breton et al., 2019). The strength of the IUCN Red List is the ability to use it to compare the extinction risk of species across all systems; without the use of strict definitions and consistent assessment methodology, this would not be possible (Collen & Böhm, 2012; Collen et al., 2016).

An approach based on our location‐threat matrix could, therefore, be applicable to other insular habitats, including terrestrial and freshwater systems, although this is limited to cases that have equivalency to the key features of the vent Red List: species endemic to a particular habitat; habitat occurs in spatially small, insular patches; limited mobility of species between habitat patches; and potential threat would cause eradication of all local habitat. This is potentially the case in many other threatened insular habitats including, for example, regions that are the targets of terrestrial mining. A high number of *Cyrtodactylus* geckos have recently been described from karstic areas in Myanmar (e.g., Grismer et al., 2018). Fourteen of these newly described species originate from only 17 karst towers over a linear distance of 100 km (Grismer et al., 2020), a distribution pattern not dissimilar to vent‐endemic molluscs. The location‐threat matrix approach can be applied to these species, especially to distinguish where quarrying for limestone is already occurring (assessments would likely follow the outcomes as per the row “mining license” in Figure 1) and where no quarrying is yet in place (row “no mining license” in Figure 1). Protection has to be effective to invoke LC assessments; this is particularly important for Southeast Asian karstic areas that are largely legally unprotected (Clements et al., 2006). Given the lucrative business of quarrying for cement production, legal karst conservation is extremely difficult (Grismer et al., 2020), akin to prevention of exploitation of hydrothermal vents.

The IUCN Red List criteria can be applied successfully and correctly to understudied taxa in a habitat entirely new to the red‐listing process. With our approach, we were able to identify and summarize the variables that most affected Red List assessment outcomes for hydrothermal vent‐endemic species in our location‐threat matrix. The location‐threat matrix translates the IUCN Red List framework into a simple, preliminary tool to evaluate the extinction risk of vent‐endemic species, a tool we also believe will provide insight to conservationists studying species in similarly insular systems worldwide.

## Supporting information

Appendix S1. Vent Red List assessments published up to 2020. IUCN Red List category abbreviations: CR, critically endangered; EN, endangered; VU, vulnerable; NT, near threatened; LC, least concern.
