# Supplementary material for: Assessing the extinction risk of insular, understudied marine species
Source: Conserv Biol. 2021 Nov 12;36(2):e13854. doi: 10.1111/cobi.13854 (PMC9299203; doi:10.1111/cobi.13854)
Supplement: Supplementary file 1 — Appendix S1. Vent Red List assessments published up to 2020. IUCN Red List category abbreviations: CR, critically endangered; EN, endangered; VU, vulnerable; NT, near threatened; LC, least concern. [file COBI-36-0-s001.docx]

**Supporting Information**

Appendix S1. Vent Red List assessments published up to 2020.2. IUCN Red List category abbreviations: CR critically endangered, EN endangered, VU vulnerable, NT near threatened, LC least concern.

| Species | Red List category | Assessment criteria | Number of locations | Mining threat | Protection | Assessment DOI |
| --- | --- | --- | --- | --- | --- | --- |
| *Alviniconcha adamantis* | EN | B1ab(iii)+2ab(iii) | 2 | Japan EEZ | No | [https://dx.doi.org/10.2305/IUCN.UK.2019-3.RLTS.T145380159A145380579.en](about:blank) |
| *Alviniconcha boucheti* | EN | B2ab(iii) | 3 | Papua New Guinea EEZ | No | [https://dx.doi.org/10.2305/IUCN.UK.2019-3.RLTS.T145380180A145380584.en](about:blank) |
| *Alviniconcha hessleri* | VU | D2 | 2 | No | No | [https://dx.doi.org/10.2305/IUCN.UK.2019-3.RLTS.T145380272A145380599.en](about:blank) |
| *Alviniconcha kojimai* | EN | B2ab(iii) | 4 | Papua New Guinea EEZ | No | [https://dx.doi.org/10.2305/IUCN.UK.2019-3.RLTS.T145380214A145380589.en](about:blank) |
| *Alviniconcha marisindica* | EN | B2ab(iii) | 4 | ISA contract | No | [https://dx.doi.org/10.2305/IUCN.UK.2019-3.RLTS.T145379801A145380574.en](about:blank) |
| *Alviniconcha strummeri* | VU | D2 | 1 | No | No | [https://dx.doi.org/10.2305/IUCN.UK.2019-3.RLTS.T145380245A145380594.en](about:blank) |
| *Bathymodiolus brevior* | VU | D2 | 3 | No | No | [https://dx.doi.org/10.2305/IUCN.UK.2020-2.RLTS.T200959A2686572.en](about:blank) |
| *Bathymodiolus elongatus* | VU | D2 | 1 | No | No | [https://dx.doi.org/10.2305/IUCN.UK.2020-2.RLTS.T201004A2688298.en](about:blank) |
| *Bathymodiolus manusensis* | EN | B1ab(iii)+2ab(iii) | 1 | Papua New Guinea EEZ | No | [https://dx.doi.org/10.2305/IUCN.UK.2020-2.RLTS.T201164A2694033.en](about:blank) |
| *Bathymodiolus marisindicus* | VU | B2ab(iii); D2 | 5 | ISA contracts | No | [https://dx.doi.org/10.2305/IUCN.UK.2019-3.RLTS.T201085A2690857.en](about:blank) |
| *Bathymodiolus septemdierum* | EN | B2ab(iii) | 2 | Japan EEZ | No | [https://dx.doi.org/10.2305/IUCN.UK.2020-2.RLTS.T201075A2690611.en](about:blank) |
| *Bruceiella globulus* | VU | D2 | 2 | No | No | [https://dx.doi.org/10.2305/IUCN.UK.2020-2.RLTS.T157703119A157703240.en](about:blank) |
| *Bruceiella indurata* | LC |  | 2 | No | SGSSI MPA, Antarctic Treaty | [https://dx.doi.org/10.2305/IUCN.UK.2020-2.RLTS.T170873188A170873251.en](about:blank) |
| *Bruceiella wareni* | CR | B1ab(iii)+2ab(iii) | 1 | ISA contract | No | [https://dx.doi.org/10.2305/IUCN.UK.2019-3.RLTS.T145337921A145338158.en](about:blank) |
| *Chrysomallon squamiferum* | EN | B2ab(iii) | 3 | ISA contracts | No | [https://dx.doi.org/10.2305/IUCN.UK.2019-2.RLTS.T103636217A103636261.en](about:blank) |
| *Desbruyeresia armata* | EN | B1ab(iii)+2ab(iii) | 1 | Japan EEZ | No | [https://dx.doi.org/10.2305/IUCN.UK.2020-2.RLTS.T157715467A157715744.en](about:blank) |
| *Desbruyeresia cancellata* | VU | D2 | 3 | No | No | [https://dx.doi.org/10.2305/IUCN.UK.2020-2.RLTS.T157715403A157715739.en](about:blank) |
| *Desbruyeresia costata* | EN | B1ab(iii)+2ab(iii) | 2 | Japan EEZ | No | [https://dx.doi.org/10.2305/IUCN.UK.2020-2.RLTS.T157715572A158153271.en](about:blank) |
| *Desbruyeresia marianaensis* | VU | D2 | 2 | No | No | [https://dx.doi.org/10.2305/IUCN.UK.2020-2.RLTS.T157715642A157715754.en](about:blank) |
| *Desbruyeresia marisindica* | EN | B1ab(iii)+2ab(iii) | 2 | ISA contract | No | [https://dx.doi.org/10.2305/IUCN.UK.2019-3.RLTS.T145380484A145380609.en](about:blank) |
| *Desbruyeresia melanioides* | VU | D2 | 2 | No | No | [https://dx.doi.org/10.2305/IUCN.UK.2020-2.RLTS.T157715686A157715759.en](about:blank) |
| *Desbruyeresia spinosa* | VU | D2 | 4 | No | No | [https://dx.doi.org/10.2305/IUCN.UK.2020-2.RLTS.T157715716A157715764.en](about:blank) |
| *Dracogyra subfusca* | CR | B1ab(iii)+2ab(iii) | 1 | ISA contract | No | [https://dx.doi.org/10.2305/IUCN.UK.2019-3.RLTS.T145337626A145338148.en](about:blank) |
| *Gigantopelta aegis* | CR | B2ab(iii) | 1 | ISA contract | No | [https://dx.doi.org/10.2305/IUCN.UK.2019-3.RLTS.T145337546A145338143.en](about:blank) |
| *Gigantopelta chessoia* | LC |  | 2 | No | SGSSI MPA, Antarctic Treaty | [https://dx.doi.org/10.2305/IUCN.UK.2020-2.RLTS.T157717977A157733544.en](about:blank) |
| *Ifremeria nautilei* | EN | B2ab(iii) | 4 | Papua New Guinea EEZ | No | [https://dx.doi.org/10.2305/IUCN.UK.2019-3.RLTS.T145380421A145380604.en](about:blank) |
| *Iphinopsis boucheti* | CR | B1ab(iii)+2ab(iii) | 1 | ISA contract | No | [https://dx.doi.org/10.2305/IUCN.UK.2019-3.RLTS.T145380559A145380614.en](about:blank) |
| *Lirapex politus* | CR | B1ab(iii)+2ab(iii) | 1 | ISA contract | No | [https://dx.doi.org/10.2305/IUCN.UK.2019-3.RLTS.T145337758A145338153.en](about:blank) |
| *Provanna cooki* | LC |  | 2 | No | SGSSI MPA, Antarctic Treaty | [https://dx.doi.org/10.2305/IUCN.UK.2020-2.RLTS.T170872509A170872605.en](about:blank) |
| *Provanna fenestrata* | EN | B1ab(iii)+2ab(iii) | 2 | Japan EEZ | No | [https://dx.doi.org/10.2305/IUCN.UK.2020-2.RLTS.T158153228A158153282.en](about:blank) |
| *Spinaxinus caldarium* | LC |  | 1 | No | SGSSI MPA, Antarctic Treaty | [https://dx.doi.org/10.2305/IUCN.UK.2020-2.RLTS.T170873445A170873537.en](about:blank) |
